# Co-production of “nature walks for wellbeing” public health intervention for people with severe mental illness: use of theory and practical know-how

**DOI:** 10.1186/s12889-020-08518-7

**Published:** 2020-04-01

**Authors:** Gill Hubbard, Catharine Ward Thompson, Robert Locke, Dan Jenkins, Sarah-Anne Munoz, Hugo Van Woerden, Margaret Maxwell, Yaling Yang, Trish Gorely

**Affiliations:** 1 Department of Nursing and Midwifery, University of the Highlands and Islands, Centre for Health Science, Old Perth Road, Inverness, IV2 3JH Scotland, UK; 2 OPENspace research centre, University of Edinburgh, 74 Lauriston Place, Edinburgh, EH3 9DF UK; 3 Partnerships for Well-Being, 33 Wells Street, Inverness, IV35JU Scotland; 4 NHS Highland, Larch House, Stoneyfield Business Park, Inverness, IV2 7PA UK; 5 Division of Rural Health and Well-being, University of the Highlands and Islands, Centre for Health Science, Old Perth Road, Inverness, Scotland, UK IV2 3JH; 6 Nursing, Midwifery and Allied Health Professions Research Unit, University of Stirling, Stirling, FK9 4LA Scotland; 7 Nuffield Department of Primary Care Health Sciences, University of Oxford, Radcliffe Primary Care Building, Radcliffe Observatory Quarter, Woodstock Road, Oxford, OX2 6GG UK

**Keywords:** Intervention development, Nature, Mental health

## Abstract

**Background:**

Interventions need to be developed in a timely and relatively low-cost manner in order to respond to, and quickly address, major public health concerns. We aimed to quickly develop an intervention to support people with severe mental ill-health, that is systematic, well founded both in theory and evidence, without the support of significant funding or resource. In this article we aim to open and elucidate the contents of the ‘black box’ of intervention development.

**Methods:**

A multidisciplinary team of seven academics and health practitioners, together with service user input, developed an intervention in 2018 by scoping the literature, face-to-face meetings, email and telephone. Researcher fieldnotes were analysed to describe how the intervention was developed in four iterative steps.

**Results:**

In step 1 and 2, scoping the literature showed that, a) people with severe mental illness have high mortality risk in part due to high levels of sedentary behaviour and low levels of exercise; b) barriers to being active include mood, stress, body weight, money, lack of programmes and facilities and stigma c) ‘nature walks’ has potential as an intervention to address the problem. In Step 3, the team agreed what needed to be included in the intervention so it addressed the “five ways to mental wellbeing” i.e., help people to connect, be active, take notice, keep learning and give. The intervention was mapped to key behavioural change concepts such as, personal relevance, relapse prevention, self-efficacy. In Step 4, the team worked out how best to implement the intervention. The intervention would be delivered over 12 weeks by members of the hospital team and community walk volunteers. Participants would receive a nature walks booklet and text messages.

**Conclusions:**

We developed a theoretically-informed, evidence-based nature walks programme in a timely and relatively low-cost manner relevant in an era of growing mental illness and funding austerity. Further research is required to test if the intervention is effective and if this approach to intervention development works.

## Background

While there is substantial investment in evaluating complex health interventions there are relatively few funding schemes for intervention development [1]. Hence, interventions risk being poorly designed and subsequently ineffective. Nonetheless, there is guidance for health researchers and practitioners on systematically developing and evaluating interventions including Medical Research Council (MRC) guidelines on developing and evaluating complex interventions, Intervention Mapping, Six Steps in Quality Intervention Development, and research steps in the development and evaluation of public health interventions [2–5]. It is argued that a systematic approach to intervention development improves intervention effectiveness [4].

Most guidelines for intervention development recommend use of a sound theoretical basis for the intervention [6]. Such theory offers potential explanation(s) of the process by which an intervention is expected to influence behaviour. That systematic approaches lead to more effective interventions appears to be based on an assumption that systematic approaches lend themselves to the development of theoretically-based interventions which, in turn, are assumed to improve intervention effectiveness. Yet, while theory undoubtedly is used to explain *why* an intervention is effective, evidence on the use and impact of theory (or theories) as a basis for improving the effectiveness of an intervention is equivocal [7].

Careful choice of behaviour change techniques (BCTs) is also recommended to improve intervention effectiveness [8]. BCTs are an intervention’s content and techniques [9]. Hence, it could be argued that systematic approaches increase the chances of interventions being effective because these approaches lead to more appropriate selection of BCTs. There is some evidence of BCTs changing behaviours and thereby improving intervention effectiveness [9]. BCTs should align with the chosen theory (ies) [7]. Taken together, theory and BCTs provide a conceptual framework to test if intervention effectiveness occurs through hypothesised pathways of theoretical mediation [10]. Another important feature of an intervention that is relatively overlooked is “forms of delivery” that include for instance, deliverer, format, materials, and setting [11]. Taken together, theory, BCTs and forms of delivery comprise an intervention’s ‘active ingredients.’

A general criticism of intervention development guidelines is that they require great know-how, technical skills and resources [4]. Over-emphasis on the importance of theory and researcher-led designs for achieving intervention effectiveness has been criticized and arguments have been made for greater acknowledgement of the importance of sound practical judgement, logic and plain common-sense during intervention development [12]. Involvement of practitioners and servicer users (a term that includes patients, carers, clients and users of health and social care public, voluntary and private sector services) may inject practical know-how, and most guidelines for intervention development recommend co-production [2–5].

### Developing a “nature walks for wellbeing” intervention

In order to address the criticisms outlined above, a multidisciplinary team of academics and health practitioners and an individual from a service user group, worked together to develop a complex intervention in 2018. The aim of the project was to develop a “nature for wellbeing” intervention for people recently discharged from a mental health hospital, evaluate its implementation, refine it, and finally conduct a full randomized controlled trial of the intervention to evaluate effectiveness. This paper focuses on the development stage and describes how the “nature walks for wellbeing” was developed. There is considerable policy and practice interest in finding affordable and cost-effective interventions to help maintain long term mental health after hospital treatment, given the growing burden of mental illness within the population [13]. Much recent research has focused on the potential for natural environments to offer therapeutic benefits at low cost [14], particularly given the considerable interest in theories linking green space or natural environments and health [15, 16]. However, there remain many practical challenges in supporting engagement with natural environments, either at population or at individual level, for those who might benefit most (e.g. those suffering socio-economic deprivation and/or rural isolation) but have little current experience of using such environments for recreation or therapeutic benefits [17]. Interventions to change behaviour in such contexts have proved challenging to evidence and are likely to require a complex approach, well-founded in theory and systematically developed by academics, practitioners and servicer users in partnership with a view to effective practical implementation.

In the face of such challenges, one approach might be only to consider interventions developed after full systematic reviews of theories, interventions and evaluations, and extensive data gathering to elicit the views of service users and different groups of health professionals in some depth. While this might be the ideal, in many real-world contexts it is difficult to find the time and resources to support such activity and there may be urgent demands for some kind of intervention to be developed quickly and at low cost, to support individuals clearly in need of help. It is in this context that we set ourselves the following objective: To quickly develop an intervention that is:

- systematic,
- well founded in theory,
- well founded in practice so that it can be feasibly delivered,
- developed without the support of significant funding and therefore necessarily low-cost,
- likely to be effective and therefore justify piloting once it has been developed.

In sum, we aimed to develop a theoretically-informed, evidence-based intervention in a timely and relatively low-cost manner. This paper describes the decision-making processes on this basis, and the development of the intervention - a “nature for wellbeing” intervention for people recently discharged from a mental health hospital. Psychiatric diagnosis and classification is complex and controversial [18]. Hence, the target group for the intervention is any individual who is recently discharged from a mental health hospital. This approach is therefore inclusive and avoids reliance of a specific diagnostic category of mental illness.

## Methods

### Design

**Fig. 1:**
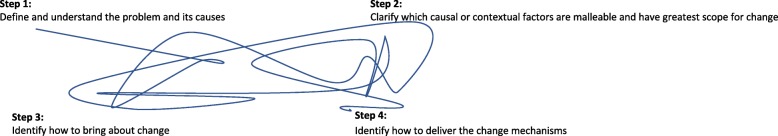
Iterative intervention development design

The MRC framework for the development of complex interventions recommend Stage I Development and Stage II Feasibility and Piloting for the development of complex interventions to improve effectiveness [2]. This article describes Stage I. How the intervention was developed aligns with the first four steps of a six step process for quality intervention development: 1. Define and understand the problem and its causes; 2. Clarify which causal or contextual factors are malleable and have greatest scope for change; 3. Identify how to bring about change: the change mechanism; 4. Identify how to deliver the change mechanism; 5. Test and refine on small scale; 6. Collect sufficient evidence of effectiveness to justify rigorous evaluation/implementation [4]. Steps 5 and 6 are dependent on securing future funding. What follows is a description of steps 1 to 4 in the process of developing the intervention and a description of the intervention itself. In this paper we present how we developed the intervention step by step and in doing so, may give the impression that we adopted a linear and sequential process to intervention development. However, it is recognized that intervention development is a more fluid and iterative activity in practice, with lots of thinking and discussion in between these steps [4, 19]. Figure 1 gives a more accurate presentation of the non-linear iterative nature of intervention development design that we followed.

### Co-producers of the intervention

Seventeen people were involved in co-producing the intervention; nine of whom are co-authors. The core team (*n* = 7) included researchers, charity representatives including a representative from a mental health service user group, and health practitioners; their expertise is presented in Table 1**.** In this manuscript we distinguish between members of the core team who were researchers (*n* = 3) and practitioners (*n* = 4).

**Table 1:** intervention developers

Researchers	1 social scientist with expertise in health services research (GH) 1 physical activity behaviour change specialist (TG) 1 landscape architect with expertise in nature-based health interventions (CWT)
Charity representatives	2 managers delivering nature-based and walking programmes (RL; SW) 1 mental health advocate (KP)
Health practitioners	1 health improvement specialist, responsible for physical activity policy (DJ)

One member of the core team (GH) acted as project manager with responsibility for keeping the momentum for intervention development, taking notes, producing descriptions of the intervention at key stages in the development process and organising meetings. The core team was supplemented with advice and support from researchers including a rural health geographer (SM), epidemiologist (HVW) health services researcher (MM), health economist (YY), three walking group volunteers, two of whom had personal experience of living with severe mental illness, and three mental health support workers (two based in a mental health hospital and one in the community).

### Setting

The intervention was designed for delivery in the Scottish Highlands, which is where the practitioners and academic project manager was based but with a view to national roll out in urban and rural locations if a future trial demonstrated effectiveness. The rate of psychiatric hospitalisation in the Scottish Highlands is 13% higher than the Scottish average of 17% [20]. A report by Mind and Scotland’s Rural College found that there were major challenges to receiving proper care in rural communities due to poor public transport and lack of support when someone with mental illness is discharged from hospital [21].

### Procedures

Intervention development began in February 2018 and ceased 12 months later. The following three main methods were used to develop the intervention in this timeframe:

1. The researchers conducted a scoping review of the literature (see Step 1 in the results section for key findings from scoping the literature) which can employ similar methods to a systematic review to provide ‘good enough’ evidence to inform the development of an intervention [22]. Literature about ‘nature and mental ill-health’ and ‘physical activity and mental ill-health’ were identified by the researchers from their own stocks of literature and from new searches in Medline which is an electronic database. The purpose of quickly scoping the literature was to identify evidence about the problems that people with severe mental illness have and to identify theories to underpin the intervention and BCTs.
2. Five two hour meetings were held between members of the research team, charity representatives and health practitioners. The purpose of the meetings was to draw on practical knowledge and expertise to inform intervention development. Hence, the meetings were designed to bring two sets of expertise – those who use research evidence and theory drawn from the literature and those who use practice know-how to develop interventions - together to make key decisions.
3. The core team communicated on a regular basis by email and telephone to comment on and refine the intervention.

### Analysis

The fieldnotes of the project manager (GH) were used to describe the co-production of the intervention, expanded by other core team member’s notes from meetings, if available. Each draft of her description of the intervention, which was based on the three sources of evidence described above, was reviewed by the core team. Any differences of opinion of what should be in the intervention were discussed and a consensus was reached.

## Results

### Step 1: define and understand the problem and its causes

The first step in intervention development in the six-step process for quality intervention development [4] is clarifying the problem and its causes.

Researchers scoped the literature to define the problem (Table 2). The international literature highlighted increased mortality risk in people with severe mental illness compared to the general population. Causal factors for increased mortality risk were that people with severe mental illness are more sedentary and less physically active than the general population. Hence, the literature suggests an urgent need for interventions to address this inequality in life expectancy. Given that we planned to pilot the intervention initially in a rural location prior to national roll out in urban and rural areas, we searched for evidence about mental ill-health in rural areas. The literature highlighted that increased mortality risk is a public health concern in remote and rural communities because in sparsely populated remote and rural areas, mental health is worse than national averages.

**Table 2:** Research team identifies the problem and its causes

Around one in four people in the UK experience a mental illness each year [13]. Mental health is generally better on average in rural areas compared to urban areas but in sparsely populated remote and rural areas, mental health is worse than national averages [23, 24]. People with severe mental illness die 15 to 20 years earlier than the general population [25]. In rural areas, this health inequality may be masked by overall and average levels of wellbeing in rural areas [26–29]. It is argued that increased mortality risk is due to the higher risk of obesity, hyperglycaemia and metabolic syndrome and subsequent development of cardiovascular disease [30]. A dual emphasis on mental and physical health is therefore essential for people with mental illness [13, 31–34] . Two potential modifiable pathways through which people’s mental illness impacts physical health and thereby mortality risk are the high levels of sedentary behaviour and low levels of exercise [35–37]. Given that people with mental illness are more sedentary and less active than the general population [35–37] and face illness-related barriers to being active [38, 39], this group needs support to become and remain more active.	

To quickly establish if an intervention to address the problem of increased mortality risk would be supported by senior health care managers, we approached two directors of public health who served urban and rural populations. The Directors for Public Health in the Scottish Highlands (HVW) and Scottish Borders, which are regions with large remote and rural populations, confirmed that ‘the problem’ identified by the researchers was a major public health concern in their regions.

### Step 2: clarify which causal or contextual factors are malleable and have greatest scope for change

The second step in intervention development is to identify factors that shape the problem and deciding where to intervene (Table 3).

**Table 3:** Factors influencing sedentary behaviour and physical activity in people with severe mental health problems

Individual	Mood Stress Body weight Money
Institutional	In-patient programmes to facilitate activities
Community	Out -patient programmes to facilitate activities Transportation
Societal	Stigma of mental illness

The researchers scoped the literature to identify factors influencing sedentary behaviour and physical activity in people with severe mental illness. A systematic review and meta-analysis from 12 studies of 6431 psychiatric patients highlighted the influence of individual-level factors; the review found that low mood and stress were the most prevalent barriers towards exercise (61% of patients) followed by lack of support (50%), and motivating factors were ‘losing weight’ (83% of patients), ‘improving mood’ (81%) and ‘reducing stress’ (78%) [39].

Other literature also highlighted community-, and institutional- level factors influencing sedentary behavior and physical activity in people with severe mental illness. The Care Quality Commission found good evidence of participation in normal day-to-day activities being made available at inpatient units, including accessing community-based walking programmes [40], but there is little evidence of provision to help people continue activities once discharged from hospital. A recent study found that inpatient rehabilitation improved activity levels but these gains were not sustained following discharge and recommended work with local community services to enable discharged patients to maintain activities [41].

In meetings, practitioners reported that there was a lack of post-discharge support in the remote and rural areas for people with severe mental illness to reduce sedentary behaviour and improve levels of physical activity. Practitioners also identified stigma of mental illness as a societal-level factor likely to influence activities, lack of public transportation to participate in community-based programmes such as walking groups, and lack of money to buy, for example, tea and cake following a group-based walk if this was a social expectation.

Practitioners reported that the mental health hospital serving remote and rural populations in the Scottish Highlands was currently working in partnership with a local charity to deliver a weekly “nature walks for wellbeing” programme (60 mins nature walk and 30 mins. Tea and cake outdoors) for in-patients. A poster produced by the charity, outlining the walks for the next 3 months, is displayed on noticeboards in the mental health hospital and in the supported housing centre. A local evaluation of the “nature walks for wellbeing” programme found that patients who went on the weekly 90 min group walk felt calmer, happier, more energetic and less tense and more relaxed; patients also said they would like to continue activities related to the programme themselves or with others after discharge from hospital [42]. Hence, the core team agreed to develop a “nature walks for wellbeing” intervention for recently discharged patients. It was proposed that a future nature walks intervention for people with severe mental illness discharged from a mental health hospital would need to address factors influencing sedentary behaviour and physical activity listed in Table 3.

### Step 3: identify how to bring about change: the change mechanism

The aim of Step 3 is to describe how a proposed intervention brings about the desired outcomes and articulate underpinning theories and active ingredients of the intervention.

The theoretical basis for a future nature walks intervention for people with severe mental illness discharged from a mental health hospital was identified from the literature. The researchers scoped the literature for supporting evidence about the potential benefits of nature walks for people with severe mental illness. The literature highlights that there are several theoretical hypotheses that have been proposed to explain how exposure to the natural environment may impact *mental* wellbeing [43, 44], including the “Biophilia hypothesis” [45, 46], Attention Restoration Theory (ART) [47] and “Psycho-evolutionary stress reduction theory” [48]. A recent systematic review of 5 systematic reviews and 50 individual papers suggests that walking benefits mental health [49]. There is also empirical evidence, summarised in systematic reviews, that being physically active outdoors has value-added mental health benefits to being active indoors [50, 51]. However, to address the problem of increased mortality risk in people with severe mental ill-health, we also needed to identify how a nature walks intervention would improve *physical* health.

The primary means by which nature has the potential to improve physical health in people with severe mental ill-health is indirectly, with nature providing a context for physical activity. As highlighted in Table 2, it is argued that increased mortality risk is due to the higher risk of obesity, hyperglycaemia and metabolic syndrome and subsequent development of cardiovascular disease [30]. There is strong evidence that small increases in physical activity is associated with reduced cardiovascular disease. A Cochrane systematic review and meta-analysis of 43 studies involving 3476 participants reported that physical activity is associated with improved cardiovascular disease risk factors in adults with overweight or obesity [52]. A systematic review and dose-response meta-analysis of cohort studies of 18 studies involving 76,699 participants reported that any amount of leisure-time physical activity is better than none and is associated with a reduction in metabolic syndrome [53]. A systematic review and meta-analysis of 36 studies involving 3,439,874 participants reported that the greatest effect of physical activity on cardiovascular disease incidence and mortality is moving from inactivity to small amounts of physical activity [54]. Several studies have been conducted about physical activity in people with severe mental ill-health. Systematic reviews suggest that physical activity is an effective treatment for depression [55, 56], schizophrenia [57], and anxiety [58] and is associated with improved anthropometric measures, aerobic capacity, and quality of life among people with mental illness [56]. There is also good evidence that physical inactivity is predictive of a range of adverse health outcomes including obesity, diabetes and medical co-morbidity among people with severe mental disorders [59–61]. Finally, we searched the literature to determine if a 12 week nature walks intervention would promote behaviour change and maintenance. We could not find research evidence specially in people with severe mental ill-health; however, there is evidence from a systematic review and meta-analysis that physical activity interventions lead to behaviour change that is sustained after 6 months or more [62].

In meetings, practitioners identified the active ingredients of the current “nature walks for wellbeing” programme being delivered in the Scottish Highlands to hospital in-patients that they believed improved physical and mental health outcomes in people with severe mental illness. The programme was group-based, involving a group of in-patients and walk leader. Tea and cake was provided free of charge after the walk. Practitioners reported that these two elements of the programme – group-based, tea and cake - provided opportunities for social interaction which they believed was important for mental health. The programme involved walking for 60 min which was perceived to yield important mental and physical health benefits. The walk took place outdoors in nature; practitioners believed that exposure to nature was restorative. The programme was delivered by people with skills in both mental illness and stigma, and knowledge about the natural environment (e.g., bird and flower identification). The same people led the walks each week, which was believed to help build relationships and rapport with the client group.

These active ingredients of the nature walks programme for in-patients were then mapped by the core team onto the “five ways to mental wellbeing” [63], which is an evidence-based framework for improving mental well-being recommended by the NHS (Table 4). The plan was to ensure that these five ways would be incorporated into a future nature walks intervention for people with severe mental illness who were discharged from a mental health hospital.

**Table 4:** Mapping active ingredients of a current “nature walks for wellbeing’ programme for in-patients to five ways to improving mental well-being

Connect	Participants join a group-based walking group, thereby potentially connecting with others during the walk and for tea and cake afterwards
Be active	Participants engage in a physical activity – walking for 60 min
Take notice	Participants are exposed to, and encouraged to observe, nature while walking outdoors, they are with a walk leader to point out things of interest
Keep learning	Participants have the opportunity to learn new things about nature from a knowledgeable walk leader who prepares the walk.
Give	Participants have the opportunity to support each other – thereby giving – and also encouraging others to participate

Generic literature about changing behaviour was also used to inform the future intervention. National Institute for Health and Care Excellence (NICE) guidance on behaviour change concluded that evidence did not support any particular models and, for this reason, recommended that behavioural change interventions focus on generic competencies and skills, rather than specific models [64]. NICE guidance recommended use of ten concepts drawn from the psychological literature to structure and inform behaviour change interventions. The core team examined these concepts with reference to factors influencing sedentary behaviour and physical activity in people with severe mental health problems (Table 3) and the five ways to improving mental well-being (Table 4); those that were deemed relevant for the planned intervention were selected. How these concepts informed the future intervention is described in Table 5.

**Table 5:** Mapping behaviour change concepts to a future nature walks intervention for people discharged from a mental health hospital

Outcome expectancies	A lay summary of evidence (see Table 1) about the benefits of nature walks will be given to people recently discharged from a mental health hospital to help them develop accurate knowledge about the health consequences of participating in the intervention
Positive attitude	Information provided to participants will aim to promote positive feelings towards the outcomes of nature walks
Personal and moral norms	Participants will be given a diary to self-monitor their participation in the group-based nature walks.
Personal relevance	Participants will be encouraged to articulate what they hope to achieve from participating in the intervention. The literature for instance, suggests that these may include reducing stress, improving mood and losing body weight.
Individual, targeted information	Participants will be given information about a local walking group in the community where they will live once they are discharged from a mental health hospital. Maps of their local area will be given should they wish to walk on their own or with a family member or friend. Participants will be supported to identify local walk routes.
Relapse prevention	Participants will identify personal barriers (e.g. low mood, stress, stigma – see Table 2) to participating in a local community walking group and be shown how to develop action and coping plans for times when these barriers feel insurmountable.
Intention formation and concrete plan	Participants will set personal and incremental goals to participate in nature walks and be supported to achieve these goals.
Self-efficacy	Participants will be encouraged to describe their experiences of nature walks with a view to encouraging self-belief in their abilities to participate in group-based walks and walking on their own
Subjective norms	Participant’s family members, friends, GP and/or mental health team will be informed about the participant’s involvement in the intervention and asked to encourage participation

Behavioural contracts (one of the ten concepts highlighted by NICE), which involve asking people to share their plans and goals with others was not chosen because the core team believed that imposing a contract would be a barrier to people with mental health problems participating in the intervention.

### Step 4: identify how to deliver the change mechanisms

The aim of Step 4 is to work out how best to implement the intervention in ‘real-world’ settings.

In meetings, the core team decided on the form of delivery, including the deliverers, materials and procedures.

#### Deliverers

There will be two intervention deliverers:

1. A member of the hospital care team (e.g., occupational therapy assistant, support worker, nurse) will meet a patient who is about to be discharged from hospital. The purpose of the meeting(s) is to plan and support the patient’s participation in nature walks once discharged from hospital and living in the community. The booklet described below under the section ‘materials’ will be used for this purpose.
2. Volunteer walk leaders from existing local community walking groups will support people recently discharged from a mental health hospital participate in local nature-based walks. The walk leaders will receive brief mental health awareness training such as, the ‘mental health awareness training for leisure staff and volunteers’ which is an online module taking around 45 min to complete and produced by the Scottish Association of Mental Health. The purpose of the training is to help increase walk leaders’ understanding of mental health and build up their confidence to welcome and support walkers affected by mental ill-health. Volunteer walk leaders will also attend at least one of the in-patient “nature walks for well-being” sessions currently delivered in the Scottish Highlands to increase familiarity about mental health issues and build confidence to support people with mental health problems attending the walks that they lead.

#### Materials

There will be two types of materials: booklet and text messages.

A “nature walks” booklet will be given to each patient before they are discharged from hospital. A member of the hospital care team and patient will work through the booklet together. Contents will include:

1. Lay summary of the benefits to physical and mental health of nature walks.
2. Section to write down personal goals relating to participating in a local community walking group.
3. Details of existing local community walking groups, including the name and contact details of the walk leader.
4. 12 week diary to record: a) date, time and location of a local community walking group; b) record when they participated in a nature walk.
5. Section to plan transportation to participate in the walk (this may include contacting voluntary transport services).
6. Section to identify potential barriers to participating in the local community walking group and writing down coping plans to manage these barriers.
7. Section to identify people to provide ongoing support and encouragement to participate in the local walking group. This may involve family and friends but also contacting a Befriender service.
8. Section to record (visually or in writing) observations while participating in nature walks.

One automated text message per week will be sent to patients for 12 weeks. The messages will be used to emphasise the benefits of nature walks and identify common barriers experienced by people with severe mental illness with tips to address these barriers.

#### Intervention procedure

The intervention will include three key procedures:

1. A member of the hospital care team will meet a patient before they are discharged from hospital and support them to complete key sections of the booklet and explain the purpose of the text messages. At the end of the 12 week programme, the patient will meet with the member of the hospital care team to discuss progress.
2. A volunteer walk leader who has already taken the online module for mental health awareness will contact the patient, inviting them to participate in the walking group, and give the patient the time and place of the next nature walk. It is recognised that contacting by phone may be problematic so a range of communication will be used including email, letter by post and telephone.
3. The patient will participate in walk in nature independently but also as part of a local walking group during the 12 week intervention. Some patients may request being accompanied by a member of the hospital care team for the first walk and every effort, taking into consideration resource constraints, will be made to facilitate this request. During the 12 week period, participants will receive text messages and also complete the diary.

#### Risks to implementation

The following risks to implementation of the intervention and potential solutions, were identified by practitioners (Table 6).

**Table 6:** Risks to intervention implementation and potential solutions

**Risks**	**Potential solutions**
Discharging of patients can be very quick, giving little time to initiate the intervention while the patient is in hospital	Hospital management consent to a member of the hospital care team visiting the patient in their own home
Capacity issues around hospital care team availability to initiate the intervention while the patient is in hospital and if required, accompany the patient on the first walk	Hospital management agree that the intervention is a priority
Volunteer walk leaders are not interested in participating in the intervention	The charity responsible for the volunteer walking group programme will consult with volunteers before the intervention is rolled out and identify those who are interested
Personal boundaries are not clearly understood	Discussions about personal boundaries with volunteer walk leaders and patients will take place. For example, it will be made clear that the walk leader will only make contact with a patient to describe each weekly walk
Poor transportation in remote and rural areas making it potentially difficult for patients to participate in local walking groups	Contact voluntary transport services
Capacity issues around befriender services in remote and rural areas	None identified

## Discussion

This paper describes how a “nature walks” intervention to improve physical and mental health in people with severe mental illness was co-produced by researchers and practitioners with input from people with experience of mental ill-health, using theory and practical know-how. By making explicit the process, we add to a growing literature reporting how interventions have been developed and contribute to opening and elucidating the contents of the ‘black box’ of intervention development [19].

There was no funding support per se to develop the intervention although our respective organisations (universities, charities, health services) endorsed our time being used for the purposes of intervention development. In this sense, the work was not cost-neutral. Limited resource influenced how much evidence and data we could gather to inform the development of the intervention but whether more labour- and resource-intensive interventions are more effective is unclear.

Like other intervention developers, reviews of qualitative and quantitative literature provided a starting point [65, 66]. We were only able to scope the literature (which included a number of relevant systematic reviews), whereas other development teams have conducted project-specific, targeted systematic reviews to understand ‘the problem’ and identify causes and causal factors [66]. How important systematic reviews are for developing interventions and increasing the chances of an intervention being effective is unclear; scoping in the early phases of intervention development may be “good enough”. In our case, we believed that we had sufficient evidence from quickly scoping the literature in combination with practitioners’ real-world experiences to understand the problem and its causes.

The intervention was co-produced by researchers and practitioners, thereby ensuring that the intervention was grounded in the ‘real world’. Other development teams using intervention mapping guidance have carried out extensive data gathering to elicit the views of patients, carers and different groups of health professionals but reported that the overall process was time-consuming and resource-intensive [66, 67]. One development team carried out in-depth interviews and qualitative thematic analysis to influence intervention development [68]. It is unclear how much data might be considered sufficient and whether more data and extensive input from multiple stakeholders, as undertaken by such teams, ultimately equates to more effective interventions.

Further, it may be more cost-effective to initially produce an intervention that does not involve extensive data collection, followed by a small-scale evaluation of its implementation before embarking on more extensive stakeholder engagement. While extensive data collection and stakeholder engagement prior to development of an intervention may be the ideal, it may not always be required or cost-effective at this stage and may be more relevant at different stages of the intervention development pathway. An advantage of producing a relatively rapidly developed intervention in the early stages is that it may avoid what Hoddinott has called premature conceptual closure [19] since nobody is expecting a definitive version of the intervention in the early development phase. Further adjustments with the intervention and challenging critical assumptions in the earlier stages [19] is possibly more acceptable and easier to take on board if the pilot intervention is presented as requiring further development. Moreover, it may be more challenging changing an intervention where people have invested so much time and effort and resource in developing it.

The intervention we developed was co-produced by researchers and practitioners in order to develop an intervention based on theory and practical know-how in a low-cost and relatively rapid way. Like other intervention developers, we did not wish the intervention to be overly “academic” or solely theory-driven [65]. There are different ways of co-producing interventions. We developed a small core team of researchers and practitioners where equal weighting was given to the respective different knowledges (theory and know-how) to shape the intervention. Other approaches to co-production include, for example, researcher-led approaches with some input from a Patient and Public Involvement consultation group [65], and extensive stakeholder involvement via mixed methods including a Patient and Public Involvement consultation group, focus groups and interviews [66]. Whether one approach yields a different type of intervention or a more effective intervention is unknown but we suggest our project, as described here, makes a contribution to considering these issues and, if the project is implemented (as we hope it will be), to testing some of principles that might underlie development of effective and cost-effective complex interventions.

### Limitations

In this article we describe how we developed a complex intervention. How we did this is in keeping with guidance for intervention development [4]. The description of intervention development reported here broadly complies with the reporting recommendations for intervention description in TiDieR [69]. Nevertheless, we did not conduct an evaluation of intervention development in its own right and did not measure or test different guidance. Moreover, we are unable to assess whether different intervention development approaches yield different or more effective interventions. In summary, our project is limited to describing how the intervention was developed rather than evaluating the impact of the particular approach that we used to develop the intervention on intervention effectiveness. As part of the intervention development process we carried out a scoping review of relevant literature, which is a rapid appraisal of the literature - a ‘quick’ alternative to undertaking a systematic review. Thus, it is possible that relevant literature was missed.

### Future directions

We recognise the need for further input from people with mental ill-health (as highlighted in the section ‘co-producers of the intervention’ we included three people with experience of mental ill-health in this phase of developing the intervention) to develop the intervention and are currently addressing this by working in partnership with the Scotland Mental Health Research Network. This network includes a group of eight people with direct and personal experience of mental ill-health. We also recognise the need for more involvement by frontline practitioners (as highlighted in the section ‘co-producers of the intervention’ we included three mental health support workers of which two were based in a mental health hospital and one in the community) in helping to identify pragmatic considerations for example, hospital staff capacity to support intervention implementation and patient-level factors influencing participation such as, side effects of medication. The intervention was developed for urban and rural settings and the practicalities of implementing in these different locations may differ and hence, further research is needed to assess the feasibility and acceptability of the “nature walks for wellbeing” intervention in these two settings. Moreover, the involvement of locally-specific voices for each setting and potentially adjusting the intervention in light of their input is also likely to be necessary. Hence, the further research we are planning will include a mixed methods evaluation of its implementation in different geographical and demographic contexts and assessing the potential for intervention effectiveness and the hypothesised pathways of theoretical and technical mediation. If this next stage of the intervention development is successful, we will then apply for funding to conduct a full trial to measure effectiveness, thereby completing all six steps for intervention development [4].

## Conclusions

Multi-disciplinary and multi-role co-production enabled use of theory and practical know-how to develop a pragmatic, theoretically based “nature walks for wellbeing” intervention for people with severe mental illness. We demonstrate how this can be achieved in a timely and relatively low-cost manner relevant in an era of growing mental illness and funding austerity.

## Data Availability

All intervention materials are available from the corresponding author on reasonable request.

## Abbreviations

BCTs: Behaviour change techniques
MRC: Medical Research Council
NHS: National Health Service
NICE: National Institute for Health and Care Excellence
